# Dart Throwing with the Open and Closed Eyes: Kinematic Analysis

**DOI:** 10.1155/2019/4217491

**Published:** 2019-11-19

**Authors:** Alexey S. Smirnov, Tatiana A. Alikovskaia, Pavel N. Ermakov, Pavel P. Khoroshikh, Kirill A. Fadeev, Alexander A. Sergievich, Alexey V. Tumialis, Kirill S. Golokhvast

**Affiliations:** ^1^Regional Scientific Centre of the Russian Academy of Education, 690922 Vladivostok, Russia; ^2^Far Eastern Federal University, 690922 Vladivostok, Russia; ^3^Southern Federal University, 344006 Rostov-on-Don, Russia

## Abstract

Eye-hand coordination during dart throwing includes both the sensory and motor components, as well as cognitive variables, for example, the direction of the subject's attention to the target or to the hand kinematic. In the present study, subjects performed dart throws in the eyes-open and eyes-closed conditions with simultaneous recording of the kinematics of the throwing hand. The results showed that the position of the hand in its raising phase was closer to the torso when performing more accurate throws with the eyes-open condition compared to more peripheral throws and throws performed in the eyes-closed condition. Following the dart release, the position of the hand in the eyes-open condition was lower compared to the eyes-closed condition. Additionally, in the eyes-closed condition, raising the hand in its backward moving phase negatively predicts the throwing accuracy. Thus, the early phase of the movement is associated with attention, and the final phase is associated with the visual feedback about the throwing accuracy. Raising the hand in the eyes-closed condition reflects an increase in muscle tension, which leads to a decrease in the accuracy of movement. The results of the study can be applied in sports and in the treatment of hand-eye-coordination disorders.

## 1. Introduction

Vision makes a great contribution to the regulation of targeted hand movements. Visual information influences the formation of a movement vector [1]. For example, the visual perception of the static arm before the beginning of the movement [2–4] and the proprioceptive sensations of the hand position [1, 5] increase the accuracy of the movement. Also, hand perception in the early phase of action impacts on a more accurate motor program [6].

The visual feedback on the accuracy of movement at its end phase contributes to the increased learning rate of a dart throwing [7]. Depriving visual feedback increases uncertainty and reduces the effectiveness of the correction of the predictive motion program during the next trial [8]. van Beers [9] proposed the Planned Aim Point Correction Model, explaining the mechanisms for correcting the motor program during training based on visual feedback. According to the model, an error in achieving the final result of an action consists of the sum of a systematic planning error, noise in planning an action, and motor noise. In this model, the subject builds a plan of action based not on the result of the previous action (the relative amount of the contribution of various sources of error is unknown to the subject), but by adjusting the program of the previous action that is available to the subject for control and change. As a result, the correction of the action plan occurs only partially and amounts to about 40%. Too low a value leads to an insufficient correction; too high a percentage of correction leads to an overestimation of motor noise and a decrease in the effectiveness of training.

However, depending on the subject's task, the vision's influence on the online movement regulation varies. Some researchers believe that the role of the visual system is minimal. In particular, it was found that the time factor of the dart release [10], the position of the arm and the speed of the movement [11], the amount of muscle activity [12], interaction torques [13], and other parameters are important in improving the accuracy in the throwing task. In the course of the action, visual information is available for the correction with a latency of 115–147 ms [14–16]. At the same time, the time window of the release of a javelin with darts professionals is 2.1 msec, and for beginners, it is about 4.2 msec [10], which is significantly less than visual online motion control. It is likely that the movement is based on the forward model and the possibilities for visual regulation are significantly limited. In contrast, other researchers have shown the need for visual perception to correct abnormal movements of the hand caused in pointing tasks by a change in target position during movement [15], or while using lenses that change the perception of a target's location [17].

Another factor that influenced the dart throwing performance is an attentional focus. External focus of attention, which is defined as concentration on movement effects such as bull's eye on the target, provides more accurate performance of the action with smaller levels of muscle tension in various tasks, such as keeping the balance [18], long jumps [19], and darts throwing [12]. Internal focus of attention that is introduced as concentration on body movements, such as focusing on muscle tension and dynamics of the effector movement, causes a higher tension of the antagonist muscles and a decrease in the motion variability [20, 21], which leads to a decrease in the accuracy of the action [22]. According to the constrained-action hypothesis, the attempts to consciously control one's own movement hamper the motor system through interference with the motor control process, which normally regulates movement [23]. Lohse et al. [24] presented a theory of attention in motor control and considered attention as an act of improving the accuracy of the measurement of the target presentation. According to this theory, an increase in accuracy in the external focus of attention condition is associated with the increase in the motion variability and correlations among bodily dimensions, which reflects the compensatory mechanisms of the regulation of movement for nontarget measurements. In contrast, an internal focus of attention reduces the variability of movement and the accuracy of throws.

The study on the role of attention and visual information in regulating the accuracy of dart throws was done by Sherwood et al. [25]. In the second experiment, which used a two by two design, subjects performed dart throws in four conditions with attention (external versus internal) and visual feedback (with or without blindfolds) variables. The results showed that the external focus of attention and the visual feedback have independent contributions to the dart throws accuracy. Another study, conducted by Sherwood et al. [26], shows that spatial errors in darts throwing were greater in internal focus of attention when vision was not available and when subjects made judgments about task-relevant dimension (joint angle) as compared to task irrelevant dimension (respiration).

In these studies, however, only subjective assessments of attention direction and accuracy of throws were analyzed. It is not clear, therefore, how the presence of visual information is reflected in the hand kinematics and at what stages of the movement's execution the presence and absence of visual information have its effect. A single study provides the data about changes in arm acceleration and explained that it depends on the external and internal focus of attention [21]. Along the less coconstruction of muscles (agonist and antagonist), there was greater acceleration in the second half of the movement in the external focus condition relative to the internal focus condition.

In the current study, we analyzed the kinematics of the movement of throwing darts in two different conditions of visual perception. The goal of the game of darts is to hit a spot in the central region of the target (bull's eye). A typical sequence of events consists of aiming, swinging, acceleration, release of the dart, and completion of the throw followed by getting feedback about the result. In the process of performing the throws, we recorded the position and speed of the hand using motion capture sensors. Subjects performed throws of the dart in eyes-open and eyes-closed conditions. The absence of visual feedback dumped the correction of the movement program and forced the subject to direct attention inward to control the kinematics of motion based on proprioception. Also, we divided the throws in eyes-open condition onto two groups above and below the individual mean score. The aim of this separation was twofold: first, to eliminate the factor of throw success on hand kinematic by comparing the eyes-closed condition with the group of throws in eyes-open condition below the mean score; second, to compare the hand kinematic of more success and less success throws in eyes-open condition. Comparing the throws above versus below the individual mean score, we investigated the lapses of the external focus of attention, and comparing the eyes-closed condition with both groups of throws in eyes-open condition, we investigated the impact the visual information to throw performance.

We assumed that in this experiment the absence of visual information would reduce the accuracy of the throws and hence our results should repeat the data of Sherwood et al. [25]. Also, the lack of visual information would not affect the dynamics of the throw due to the limited visual control for quick movements when performing throws [10, 11] but would affect the early and late stages of the throws associated with aiming and receiving feedback. Concerning the comparison of hand movement during the more success versus less success throws in eyes-open condition, we did not have any particular predictions.

Additionally, in this study, a long period was used for the analysis, which included various phases of arm movement. Although previous studies have analyzed the accuracy of dart throwing depending on the position or speed of the hand, they were limited to a short period of dart releasing [10, 11]. The data from the point task studies show that subject's hand perception in the early and late phases of the movement has a significant impact on the accuracy of the task [2–4, 6, 15], and the presence of visual feedback when performing darts also has a positive impact on the accuracy of the throws and the speed of learning [7, 8]. These data show the influence of visual reafferentation on the kinematics of the hand movement when making dart throws.

## 2. Materials and Methods

### 2.1. Subjects

The subjects were 15 students and staff of Far Eastern Federal University. The data of the two subjects was removed due to technical errors during data registration. Hence, the final number of subjects was 13 (age = 25.08± 7.5, body mass index = 21.67 ± 3.34, number of men *N* = 8, years of education = 15.5 ± 2.20, left-handed *N* = 4). Subjects had no or little (few times in their lives) experience in playing darts and no sports activities addressed accuracy in motion (shooting, throwing, cybersport, or others). All participants were volunteers. Subjects have no neurological or mental abnormalities according to the self-report. They did not consume alcohol 24 hours before the study and tonic beverages three hours before the study. The study was conducted in accordance with the Helsinki Declaration and all subjects signed an informed consent.

### 2.2. Procedure

Upon arrival at the laboratory, the subjects completed an informed consent. Then they put on a Perception Neuron 32 motion capture suit. The sampling rate was 60 Hz. Then, there was the signal calibration of the motion capture suit. After this, a warm-up took place, during which the subjects performed 10 throws. The weight of a dart was 16.5 grams, and the target consisted of concentric black and white circles with a score of one at the periphery to 10 in the center. The target was placed at a distance of 2.37 meters and a height of 1.73 meters in accordance with the rules of the World Darts Federation (https://www.dartswdf.com/rules/).

The participants were asked to throw darts at the target in two conditions—with eyes open and eyes closed. In each condition, there were 65 throws in a series of five throws. The series followed each other (eyes-open, eyes-closed, eyes-open, etc.) always starting from the eyes-open condition. To perform a series of throws, the subject was given five darts. Between the series, the researcher removed all the darts from the target. During the throws, the researcher recorded the accuracy of hitting the darts without informing the subject on the achieved result. In the eyes-open condition, the subjects had the opportunity to see the accuracy of the throws and to correct the throwing performance. In the eyes-closed condition, the subjects should not open their eyes between throws, but only after the end of the series of throws. Thus, the subjects did not see the result of each throw, but they could hear the dart hitting the target or the wall at which the target was placed and see the result of all five throws at the end of a series.

The subjects performed throws with their leading hand. If the subject was left-handed, the data from his left hand were used in the analysis. The accuracy of hits was recorded in accordance with the target hit zones ranging from 1 to 10. If the target was not hit, the score was zero.

### 2.3. Accuracy Analysis

The analysis of the accuracy of hits was carried out for the conditions of open and closed eyes and only for throws that hit the target.

The mean value of the accuracy of hits for all throws was calculated for the eyes-open condition. Then, these throws were divided into two groups—above and below the individual mean hit score, that is, central and peripheral hits (EOc and EOp), and the number of hits and mean accuracy were calculated in both groups of throws. For the eyes-closed condition (EC), all the hits were averaged.

The division of throws in the eyes-open condition into two groups was done to compare the accuracy of peripheral hit throws in the eyes-open condition with the throws in the eyes-closed condition in order to eliminate the influence of accuracy factor on the kinematics of throwing. If the difference between peripheral hit throws in the eyes-open condition and in the eyes-closed condition is nonsignificant and both groups of throws differ from the central hit throws in the eyes-open condition (EC = EOp ≠ EOc), this would indicate the influence of the attention factor (internal versus external). If the difference between the two groups of throws in the eyes-open condition is nonsignificant and both groups differ from the throws in the eyes-closed condition (EC ≠ EOp = EOc), this would indicate the influence of the factor of visual reafferentation (absence versus presence).

We propose that in the eyes-closed condition the internal focus of attention is prevalent because the subjects have not the visual feedback and forced to attract attention to the proprioceptive data. On the other hand, eyes-open condition can induce the attraction of the attention towards the aim or lapses external focus of attention which is reflected in more or less success of throwing.

### 2.4. Kinematics Analysis

For the analysis of kinematics, the sensor on the index finger of the leading hand was chosen because it had the largest displacement amplitude. The finger displacement and speed in *OY* and *OZ* axes were exported to MATLAB. For each condition, the maximum speed of the hand was identified along the *OY* axis, that is, perpendicular to the axis of the body in the direction of the throw. This parameter was chosen because it has a clearly defined peak and reflects the event of throwing a dart. Peak identification was carried out using the built-in “findpeaks” function. A similar approach to identify the throw was used in the study by Kehoe and Rice [27]. Next, the throws were analyzed in groups in accordance with the eyes-closed and eyes-open conditions and were cut into segments from −1.667 to 0.8335 sec relative to the moment of the maximum speed of the finger movement. In the eyes-open condition, the epochs were divided into groups in accordance with the accuracy of the throw—above and below the average hit score. In the eyes-closed condition, the trajectories of all throws were analyzed. Before averaging, trajectories were checked and too deviating ones were deleted. The percentage of deleted trajectories was 1.6% for the category of central hits with eyes open, 1.7% for the category of peripheral hits with eyes open, and 2.7% for the category of hits with eyes closed. The differences between the percentages of the deleted trajectories are not significant (Friedman ANOVA Chi Sqr (*N* = 13, df = 2) = 0.21, *p*=0.902). Further, the trajectories were averaged for each test subject in each condition: central hits in eyes-open condition (EOc), peripheral hits in eyes-open condition (EOp), and hits in eyes-closed condition (EC).

When analyzing the trajectories of hand movement, we found a drift of values along the *OY* axis during the registration with 5 subjects. Therefore, to eliminate the effect of body movement on the results of localization of the hand, we calculated the average value of the position in each trial in the period from −1.5 sec to −1.167 sec and subtracted it from all subsequent values. This procedure for removing reference values was carried out along both the *OY* axis and the *OZ* axis.

Statistical analysis of the differences between the categories of throws was carried out in the SPM1d package (http://www.spm1d.org/) [28]. This package is designed for the analysis of kinematics on the basis of scripts for the analysis of three-dimensional tomography results in the SPM package. It uses random field theory (RFT), which is charged with solving the problem of multiple comparisons. Random field theory is superior to other correction methods since it conducts inference based on the height and the size of connected clusters that remain following the suitably high SPM{*t*} thresholding (e.g., *t* > 3.0). Precise probability computations additionally depend on field smoothness and search space morphology. A key point is that a large suprathreshold cluster is the topological equivalent of a large univariate *t*-value.

So, SPM1d allows analyzing trajectories for the entire duration of the movement. It also provides an opportunity to make corrections for multiple comparisons (Familywise Error Rate, FWER). Since the research is exploratory, we carried out an analysis of the long-term area of the action, without breaking it up into separate periods of analysis. This raises the threshold for Familywise Error Correction (FWEC), but the results are more resistant to accepting false alarms. In the statistical analysis, the interval from −1.167 sec to 0.5 sec relative to the time of the maximum speed of the throw was used, and it included all phases of the throw: raising the arm, swing, acceleration, throwing, and completing the throw followed by lowering the arm.

The duration of the periods between throws is a complex variable that includes the phases of the throw and the period between the throws and rather crudely indicates the activity of the regulatory mechanisms of movement performance. The duration of the periods between throws was calculated as follows: the length of the periods between the maximum speeds of the index finger of the leading hand was calculated when each throw was made within the set of throws. Then the periods above three standard deviations from the mean duration were excluded from the analysis. The remaining values were averaged by the eyes-open and eyes-closed conditions.

Nonparametric ANOVA with repeated measurements with the inclusion of three categories of throws (EOc, EOp, and EC) was used for the analysis of the effects. The significance level was calculated using permutation statistics; the number of permutations was 1000. To account for multiple comparisons, the FWER correction was used. Pairwise differences were evaluated using Student's *t*-test (with FWER correction).

Nonparametric criteria were used to analyze the data because of the small sample size. To identify the effects of a group of throws (EOc, EOp, and EC), Friedmann criterion was used. For pairwise comparisons, the Wilcoxon test was used. For the analysis of relationships, Spearman's rank correlation coefficient was used.

For the calculation of nonparametric effect size to compare paired measurements, the eta squared was used: *η*^2^ = *Z*^2^/*N*, where *Z* is the Wilcoxon *Z*-value, and *N* is the number of observations. For the calculation of nonparametric effect size to compare three paired measurements, Kendall's *W* test was used: *W* = *χ*^2^/*N*(*k *− 1), where *χ*^2^ is the Friedman ANOVA value, *N* is the sample size, and *k* is the number of measurements per subject.

The significance level was *p* < 0.05.

## 3. Results

### 3.1. Periods between Throws and Throw Accuracy

The number and accuracy of throws of each category are presented in Table 1. In the eyes-closed condition, the number and accuracy of hits were lower compared to the eyes-open condition.

Stins et al. [29] discovered the effect of a throw number in a series. To analyze this phenomenon, in the present study, an additional control analysis was performed to identify possible dynamics within a series of 5 throws. ANOVA in the eyes-closed condition showed that there is a nonsignificant effect of accuracy (Friedman Chi-square = 3.41, *p*=0.492). A similar result was obtained in the eyes-open condition (Friedman Chi-square = 3.61, *p*=0.461). The length of the periods between throws in the eyes-open and eyes-closed conditions did not differ (Wilcoxon *Z* = 1.01, *p*=0.311) and correlated at highly significant level (Spearman *R* = 0.88, *p* < 0.001).

The accuracy between the eyes-open and eyes-closed conditions has nonsignificant correlation (Spearman *R* = −0.10, *p*=0.754). Also, the number of hits in the target in eyes-open and eyes-closed conditions (*R* = 0.06, *p*=0.839) does not correlate at a significant level.

Due to the fact that the accuracy of the throws in the eyes-closed and eyes-open conditions differed, we divided the throws in the eyes-open condition into groups above and below the average accuracy for each subject and identified them as central hit and peripheral hit throws.

ANOVA with a factor of the number of throws of three groups (EOc, EOp, and EC) showed a significant effect, indicating that the number of throws with eyes closed was greater than the central hits and peripheral hits in the eyes-open condition. The latter did not differ among them (Table 2).

According to the results of ANOVA with a factor of mean accuracy in three groups of throws (EOc, EOp, and EC), a significant effect was found. Pairwise comparisons showed that the accuracy of the peripheral hit throws in the eyes-open condition did not differ from that in the throws with eyes closed, and both differ from the central hit throws in the eyes-open condition (Table 2). Thus, the accuracy of the throws in eyes-closed condition and peripheral hit throws in the eyes-open condition was aligned, and the differences in hand movement between these groups of throws cannot be associated with accuracy, but only with the presence of visual information.

### 3.2. Analysis of Hand Kinematic

**Figure 1:**
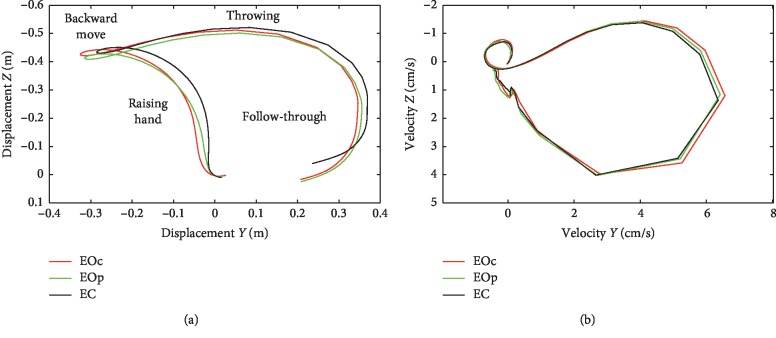
Mean displacement (a) and speed (b) in eyes-open condition with central throws (EOc), peripheral throws, and eyes-closed (EC) condition along the *OY* axis and *OZ* axis.

The movement and speed of a hand in three categories of throws are shown in Figure 1. The phases of the initial raising of the hand, backward move, acceleration, throw, and follow-through can be identified in the figure.

**Figure 2:**
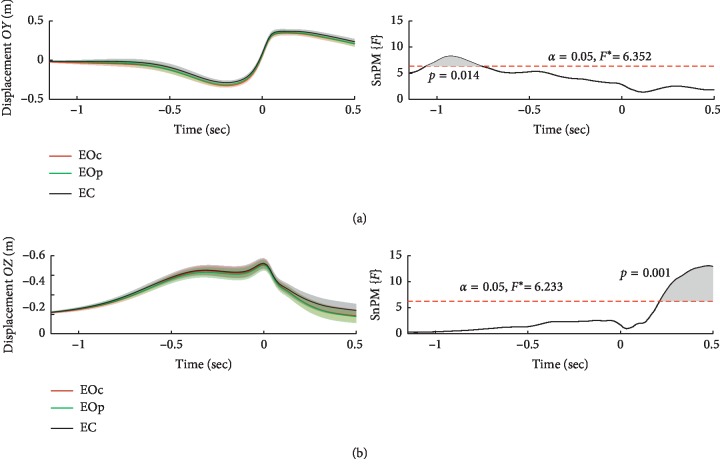
Mean ± SE displacement of three groups of throws and ANOVA effects along the *OY* (a) and *OZ* (b) axes. EOc: eyes-open condition with the central hit throws; EOp: eyes-open condition with the peripheral hit throws; EC: eyes-closed condition; SnPM{*F*}: statistical nonparametric mapping of *F*-values.

According to the movement of the hand along the *OY* axis (Figure 2(a)), that is, in the direction of the throws and perpendicular to the longitudinal axis of the body, a statistically significant effect of throws in the period from −1.058 to −0.758 seconds relative to the maximum speed of the hand was detected. This period is located in the initial phase of raising the hand and is qualified as the aiming period, that is, setting the motor program to perform a more accurate throw.

**Figure 3:**
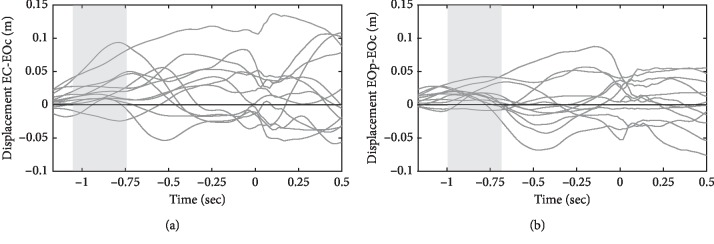
Displacement differences between EC and EOc (a) and EOp and EOc (b) along the *OY* axis in each subject. Gray rectangles depict the time periods of significant differences (*p* < 0.05).

Figure 2(a) shows mean ± SE and it could be seen that mean differences are mild due to the large individual variability.  Figure 3 shows differences between EOp and EOc as well as EC and EOc in *OY* axis. Individual traces give evidence that in the early phase the hand displacement (depicted in gray rectangles) was more positive in EOp and EC compared to EOc throws in most subjects.

**Figure 4:**
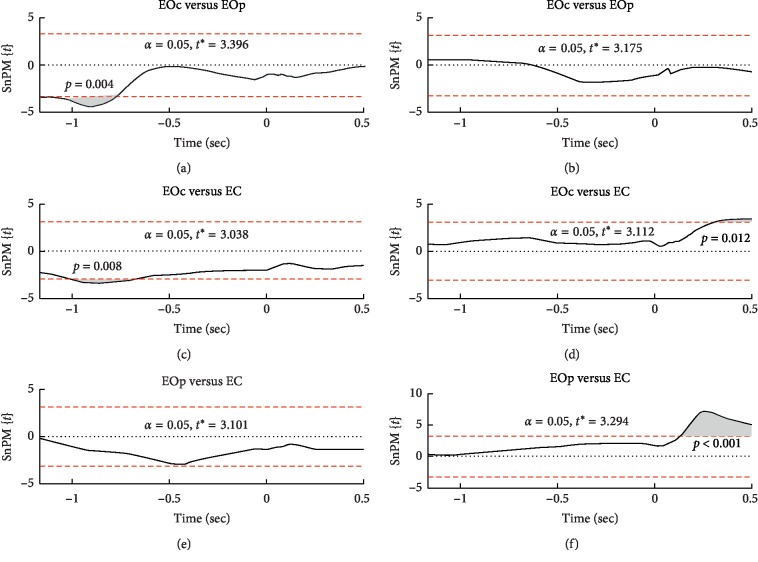
Pairwise comparisons between groups of throws along the *OY* axis (a, c, e) and *OZ* axis (b, d, f). EOc: eyes-open condition with the central hit throws; EOp: eyes-open condition with the peripheral hit throws; EC: eyes-closed condition. SnPM{*t*}: statistical nonparametric mapping of *t*-values.

Pairwise comparisons (Figure 4) showed that the trajectory of the hand in the eyes-closed condition did not differ from the movement of the hand when making peripheral hit throws in the eyes-open condition. However, more accurate central hit throws in the eyes-open condition differ from peripheral hit throws (in periods from −1.162 to −1.133 seconds and from −1.101 to −0.776 seconds) and from throws in the eyes-closed condition (in the period from −1.009 to −0.692 sec). Thus, with more accurate throws, the position of the arm in the early phase of the movement was closer to the body.

Movement of the arm along the axis *OZ*, that is, the height of the hand, has a significant effect in the period from 0.211 sec until the end of the analysis period, that is, up to 0.5 sec (Figure 2(b)). Following the dart release in the eyes-closed condition, the hand was higher compared to the eyes-open condition (OEc vs. EC: 0.302–0.5 sec; OEp vs. EC: 0.135–0.5 sec). Thus, the effect on the *OZ* axis is associated with the presence of visual information.

*OY* and *OZ* speeds did not show significant effects of the throw group.

### 3.3. Accuracy Prediction by the Hand Displacement and Speed

**Figure 5:**
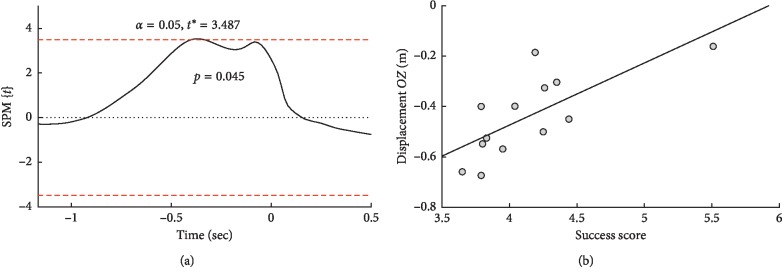
Prediction of the throw accuracy by the hand position along the *OZ* axis in the eyes-closed condition. (a) Plot of regression along the time axis. (b) Scatterplot of correlation between the throw accuracy and hand position along the *OZ* axis from −403 to −334 ms. SnPM{*t*}: statistical nonparametric mapping of *t*-values.

In the eyes-closed condition, the regression analysis between the accuracy of the throws and the position of the arm on the *OY* axis did not show significant prediction. Regression analysis between the accuracy of the throws and the position of the arm on the *OZ* axis showed that the magnitude of raising the arm on the backward move period from −403 to −334 ms predicts the accuracy of hitting the target (Figure 5). The Spearman correlation during this period (*R* = 0.73, *p*=0.004) also indicates an association. In Figure 5, it can be seen that one subject is an outlier. The removal of this subject from the analysis led to a decrease in predictive power to a subsignificant level. The correlation also decreased but remained significant (*R* = 0.66, *p*=0.019). Recalculating the ANOVA results of the differences between the groups of throws when deleting this subject showed that all effects remained significant.

Hand speeds do not predict throw accuracy. In the eyes-open condition, significant predictions of accuracy were not found.

## 4. Discussion

The experiment investigated the effect of visual information on the accuracy and kinematics of the dart throwing. It has been found that closing eyes leads to a decrease in the accuracy of throwing. The analysis of the kinematics showed that the position of the hand in the early phase of the movement was closer to the body in the eyes-open condition with more accurate throws, compared to less accurate throws in the eyes-open as well as in eyes-closed conditions. Also, following the dart release in the eyes-open condition, the hand was lower compared to the eyes-closed condition. Finally, the height of hand in backward move phase in eyes-closed condition negatively predicts the throwing accuracy.

Throwing accuracy decreased in the eyes-closed condition. It is consistent with the results of Sherwood et al. [25] and suggests that the effective performance of the motor program requires visual feedback. This conclusion does not indicate the role of visual feedback at different stages of the movement. Information on the effect of temporal parameters of motion on the accuracy of throws in this study was obtained by analyzing the kinematics during the execution of darts throws.

An additional analysis showed that the eyes-open and eyes-closed conditions do not differ in accuracy across the five throws of a series. These data contradict the results of the study conducted by Stins et al. [29], which was obtained from professional darts players. The subjects in the current study were naive and therefore less accurately corrected the program in action.

Also, in eyes-open and eyes-closed conditions, the duration of the periods between throws does not differ and was highly correlated between the conditions. Periods between throws include both a throw and a pause between throws. Performing movements is quite stereotypical and varies little in time; consequently, changes in this parameter can be largely caused by differences in the duration of pauses between throws. In particular, the subjects had periods of 2.3 to 4.6 seconds, while the execution of the throw was approximately 1.5 seconds. Thus, the preparation for a dart throw takes equal time in both the eyes-open and eyes-closed conditions. However, the accuracy and the number of hits do not have significant correlations between the eyes-open and eyes-closed conditions, reflecting a significant difference in the movement programs.

Some studies [12, 20–22, 30] found that the external focus of attention is associated with higher values of accuracy and internal focus of attention reduces its efficiency through greater movement restriction. In the current study, the position of the hand was closer to the torso in the early phase of movement when starting a more accurate throw reflects the automatic movement regulation and external focus of attention. A decrease in accuracy of throws occurred when the position of the hand was farther from the body in the early phase of movement which is associated with an arbitrary aiming and control of the movement, reflecting the activation of internal focus of attention to proprioceptive sensations. These results are consistent with the earlier data [2–4] which found that the early perception of a static hand before the pointing task was associated with a later precise movement. However, in contrast to these results, in the present study, more accurate throws were made when the hand was positioned closer to the body and therefore it is less likely that the arm came into view when it was raised. Thus, motor regulation is based primarily on proprioceptive data. Otherwise, inaccurate throws with eyes open are performed with a greater distance of the arm from the body with a greater probability to get into the field of view and so increase the visual control of the movement. But this hypothesis is not supported by the data of eyes-closed condition. In this condition, visual information was not available, but hand position was also farther from the torso as for less accurate throws in eyes-open condition. Alternatively, the attention factor may explain the current results. In the eyes-closed condition, motor regulation was based on visual working memory and online proprioceptive data. So, a greater distance of the hand from the body in the absence of visual information may occur if the subjects tried to effectively control the movement in order to reach a greater performance, based on visual image stored in the working memory. In the eyes-open condition, in the absence of additional sensory stimulation, less accurate throws occur due to the lapses of external focus of attention and/or directing the attention to the aiming, i.e., movement control based on greater impact of proprioceptive data. We suggest that farther hand position from the torso in less accurate throws in the eyes-open condition also draws greater attention to the motor and proprioceptive data, which is the internal focus of attention, due to the aiming in order to control the movement and increase throw success. Contrastingly, throws with greater accuracy in the eyes-open condition executed with the less distance of the hand from the torso reflect more automatic motor control in the external focus of attention. These interpretations are more consistent with the constrained-action hypothesis [23], according to which, drawing attention to the limb reduces the variability of the regulation of movement and, as a consequence, decreases accuracy.

The results showed the absence of significant differences in the realization of the dart throw around the peak speed of the hand, indicating that the execution of the movement under these conditions is automatic in accordance with the initialized program. These results are consistent with the notion that the feedback of the visual system is too slow for the motor program to be corrected during the course of the fast movement [14–16].

However, in the time period after the throw, the position of the hand in the eyes-closed condition was higher compared to both throw groups in the eyes-open condition. During this period, the visual feedback on the accuracy of the throw was obtained and the visual reafferentation in this phase plays a significant role in correcting the follow-up program [7, 8]. The current study additionally analyzed the hand kinematics, which showed that deprivation of visual information resulted in a higher hand position after the dart was released. The lack of visual feedback forces the subject to utilize other sources of information, for instance, auditory and somatosensory. Both sensory modalities carry only a limited amount of data and therefore the subject performs longer cognitive processing, reflected in higher hand position, and a small degree of movement freezing.

To what extent does the movement and speed of the hand predict the accuracy of the throw? Results by Nasu et al. [10] indicate the influence of the position of the hand and the release time of the dart on the throw accuracy; Smeets et al. [11] discovered the effects of hand speed on the accuracy of dart throws. However, both studies analyzed a very limited portion of the time and position of the release of the dart from the hand in the condition of undisturbed visual-motor coordination. In the present study, we analyzed all the trajectories of hand movement when making throws in the condition of open and closed eyes. The results showed that in the eyes-open condition neither the position of the hand nor the speed of the hand predicts the accuracy of the throw. In the eyes-closed condition, a higher position of the arm during a backward move leads to less accurate throws. A higher arm position requires more effort to lift and hold it. As a result, an increase in muscle tension leads to a reduction in the mobility of the hand and a decrease in the accuracy of movement [20, 21].

Our study has limitations. First, the conclusions about the impact of attention based on the throw accuracy and hand kinematics require verification in studies using the attention modulation paradigm. Also, in future studies, it will be possible to use eye tracking to determine the direction of gaze during preparation and the initial phase of the throws. Second, the results of this study are based on the analysis of the kinematics of one part of the body, namely, the index finger of the leading hand. Although similar restrictions are present in other works, for example, [29], data analyses from other parts of the limb are also required. Third, we did not register the sector of dart position at a board and, therefore, we have no information about the relative error of the throws. With the availability of this information, the regression of the vertical position and the speed of the hand on the vertical deviation of the accuracy of throws would be more adequate.

## 5. Conclusions

In the present experiment, we studied the hand kinematics of dart throwing in the eyes-open and eyes-closed conditions. It was found that more accurate throws in the eyes-open condition were made when the hand was raised closer to the body, and less accurate throws in the eyes-open condition and throws with eyes closed were made with a larger distance of the hand from the body, reflecting the external and internal focus of attention, respectively. At the final phase of the throwing, a higher hand position in the eyes-closed condition compared with the eyes-open condition was found, reflecting the extended cognitive processing of the limited information about throwing accuracy. Furthermore, raising the hand in the eyes-closed condition predicts dart throwing accuracy. We suggest that the amount of lifting the hand is associated with muscular effort and limits the lability of the regulation of movement.

## Figures and Tables

**Table 1:** Accuracy and number of throws (mean and standard deviation) for different categories of throws.

	EC	EO	EOp	EOc
Success	4.14 (0.48)	5.59 (0.69)	3.70 (0.77)	7.25 (0.46)
*N*	47.54 (6.49)	61.46 (3.21)	33.0 (5.32)	28.46 (4.45)

**Table 2:** Statistical results for different categories of throws.

		EC vs. EO^*∗*^	EOc vs. EOp^*∗*^	EC vs. EOc^*∗*^	EC vs. EOp^*∗*^	EC vs. EOc vs. EOp^*∗∗*^
Success	Effect	3.11	3.18	3.18	1.36	21.39
	*p* value	0.002	0.001	0.001	0.173	<0.001
	Effect size	0.37	0.39	0.39	0.07	0.82
*N*	Effect	3.18	1.61	3.18	3.18	20.46
	*p* value	0.001	0.107	0.001	0.001	<0.001
	Effect size	0.39	0.10	0.39	0.39	0.79

*Notes*. ^*∗*^Wilcoxon test and *η*^2^ as effect and effect size. ^*∗∗*^Friedman ANOVA and W as effect and effect size.

## Data Availability

No data were used to support this study.

## Supplementary Materials

Supplementary Materials Figure 1: displacement differences between EOc and EC (A) and EOp and EC (B) along the *OZ* axis in each subject. Figure 2: hand movement trajectories along the *OY* axis during the dart throwing in 13 subjects. Figure 3: hand movement trajectories along the *OZ* axis during the dart throwing in 13 subjects.
