# Supplementary material for: Dart Throwing with the Open and Closed Eyes: Kinematic Analysis
Source: Comput Math Methods Med. 2019 Nov 19;2019:4217491. doi: 10.1155/2019/4217491 (PMC6885837; doi:10.1155/2019/4217491)
Supplement: Supplementary Materials — Figure 1: displacement differences between EOc and EC (A) and EOp and EC (B) along the OZ axis in each subject. Figure 2: hand movement trajectories along the OY axis during the dart throwing in 13 subjects. Figure 3: hand movement trajectories along the OZ axis during the dart throwing in 13 subjects. [file 4217491.f1.pdf]

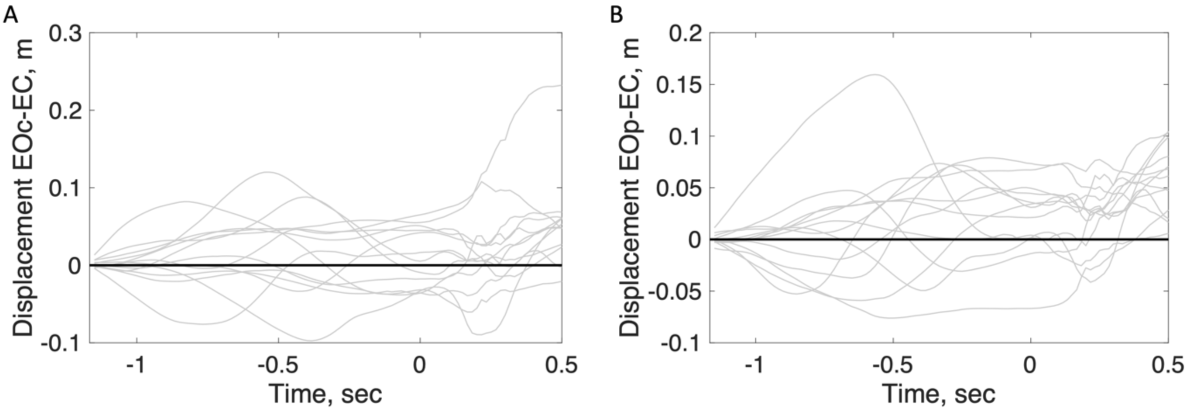

Figure 1. Displacement differences between EOc and EC (A), EOp and EC (B) along the OZ axis in each subject.

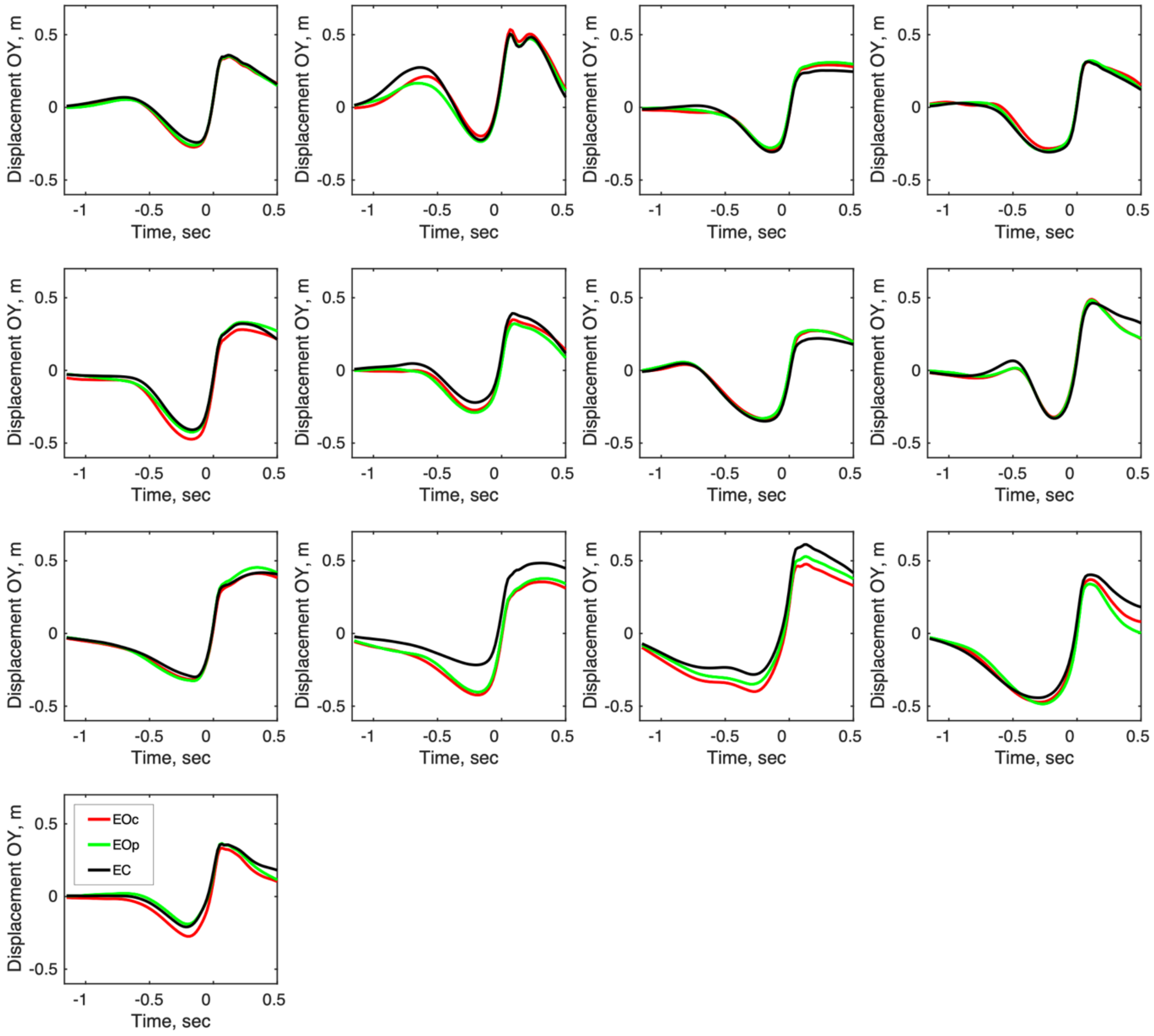

Figure 2. Hand movement trajectories along the OY axis during the dart throwing in 13 subjects.

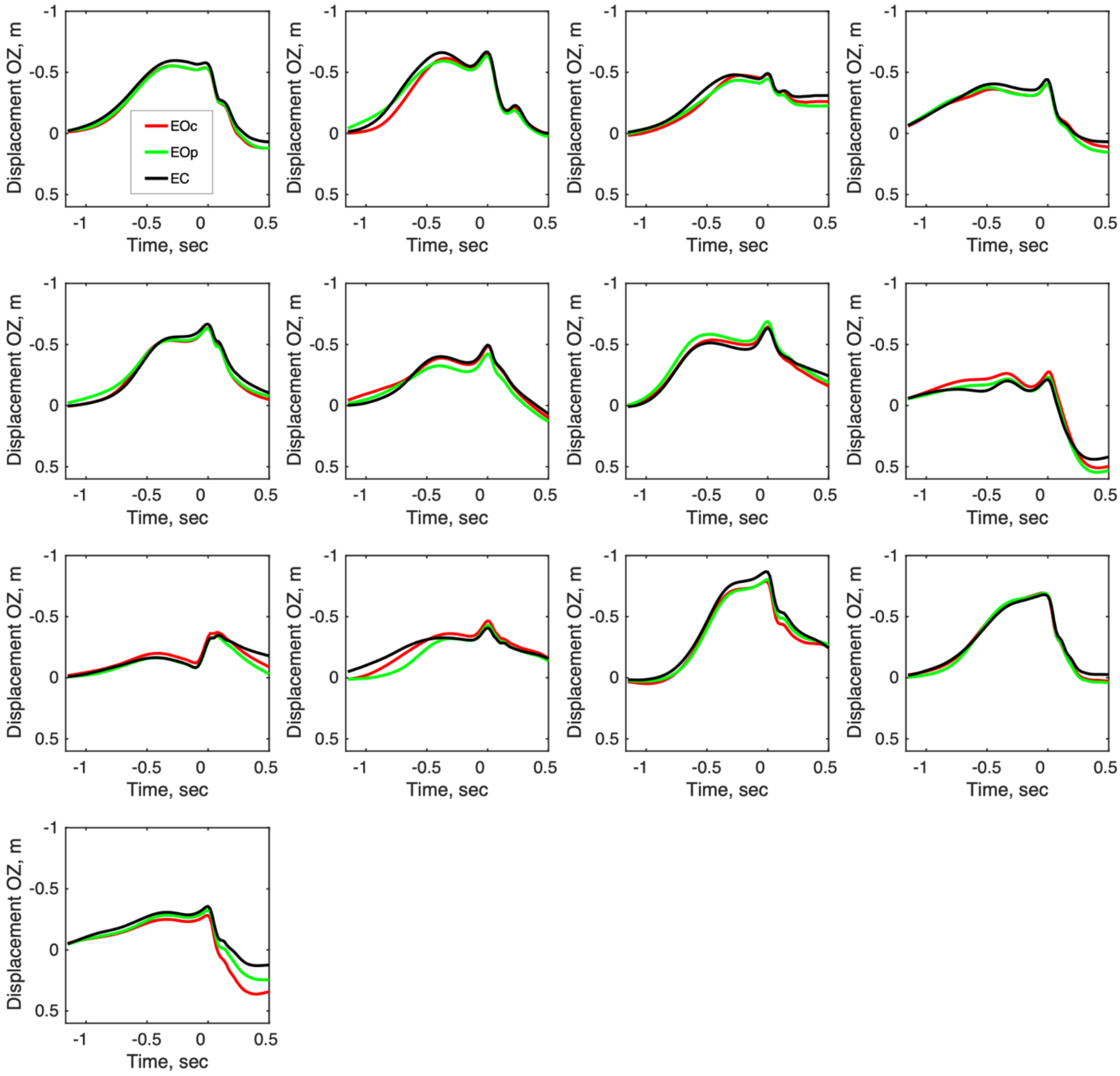

Figure 3. Hand movement trajectories along the OZ axis during the dart throwing in 13 subjects.
